# Long-term activation of anti-tumor immunity in pancreatic cancer by a p53-expressing telomerase-specific oncolytic adenovirus

**DOI:** 10.1038/s41416-024-02583-0

**Published:** 2024-02-05

**Authors:** Masashi Hashimoto, Shinji Kuroda, Nobuhiko Kanaya, Daisuke Kadowaki, Yusuke Yoshida, Masaki Sakamoto, Yuki Hamada, Ryoma Sugimoto, Chiaki Yagi, Tomoko Ohtani, Kento Kumon, Yoshihiko Kakiuchi, Kazuya Yasui, Satoru Kikuchi, Ryuichi Yoshida, Hiroshi Tazawa, Shunsuke Kagawa, Takahito Yagi, Yasuo Urata, Toshiyoshi Fujiwara

**Affiliations:** 1 Department of Gastroenterological Surgery, Okayama University Graduate School of Medicine, Dentistry and Pharmaceutical Sciences, Okayama, Japan; 2 Minimally Invasive Therapy Center, Okayama University Hospital, Okayama, Japan; 3 Center for Innovative Clinical Medicine, Okayama University Hospital, Okayama, Japan; 4 Clinical Cancer Center, Okayama University Hospital, Okayama, Japan; 5 Oncolys BioPharma, Inc., Tokyo, Japan

**Keywords:** Cancer immunotherapy, Immunotherapy

## Abstract

**Background:**

Pancreatic cancer is an aggressive, immunologically “cold” tumor. Oncolytic virotherapy is a promising treatment to overcome this problem. We developed a telomerase-specific oncolytic adenovirus armed with p53 gene (OBP-702).

**Methods:**

We investigated the efficacy of OBP-702 for pancreatic cancer, focusing on its long-term effects via long-lived memory CD8 + T cells including tissue-resident memory T cells (TRMs) and effector memory T cells (TEMs) differentiated from effector memory precursor cells (TEMps).

**Results:**

First, in vitro, OBP-702 significantly induced adenosine triphosphate (ATP), which is important for memory T cell establishment. Next, in vivo, OBP-702 local treatment to murine pancreatic PAN02 tumors increased TEMps via ATP induction from tumors and IL-15Rα induction from macrophages, leading to TRM and TEM induction. Activation of these memory T cells by OBP-702 was also maintained in combination with gemcitabine+nab-paclitaxel (GN) in a PAN02 bilateral tumor model, and GN + OBP-702 showed significant anti-tumor effects and increased TRMs in OBP-702-uninjected tumors. Finally, in a neoadjuvant model, in which PAN02 cells were re-inoculated after resection of treated-PAN02 tumors, GN + OBP-702 provided long-term anti-tumor effects even after tumor resection.

**Conclusion:**

OBP-702 can be a long-term immunostimulant with sustained anti-tumor effects on immunologically cold pancreatic cancer.

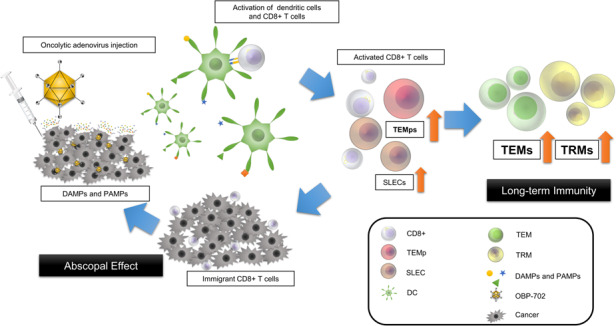

## Background

Pancreatic cancer is one of the most aggressive cancers, and its prognosis is still extremely poor despite the development of various multimodal treatment strategies [1]. Chemotherapy is currently a standard treatment even for resectable and borderline resectable pancreatic cancer as neoadjuvant chemotherapy (NAC), to say nothing of metastatic or locally advanced unresectable pancreatic cancer, and gemcitabine (GEM) plus nab-paclitaxel (nab-PTX) (GN) is one of the main regimens for such cases [2]. Although immunotherapy such as immune checkpoint inhibitors (ICIs) has been developed for pancreatic cancer as for other types of cancer, it has shown little effect because pancreatic cancer is an immunologically “cold” tumor [3]; therefore, development of immunostimulants that can convert “cold” to “hot” is a desperate need [4].

Oncolytic virotherapy (OV) has emerged as a new class of cancer therapeutics, and is highly anticipated as a systemic immune stimulator [5, 6]. We developed telomerase-specific oncolytic adenoviruses (OAs), including suratadenoturev (OBP-301) which has been tested in some clinical trials including the combination with anti-PD-1 antibody [7, 8]. In a preclinical study using an OBP-301 variant for mice (OBP-502), we showed that OBP-502 local treatment strongly induced immunogenic cell death (ICD), leading to recruitment of CD8 + T cells into tumors and provided strong anti-tumor effects [9]. However, the effects of OBP-301 and OBP-502 were insufficient for aggressive tumors such as pancreatic cancer. To overcome this hurdle, we developed another OBP-301 variant armed with p53 tumor suppressor gene (OBP-702) [10]. In fact, OBP-702 had stronger cytotoxic activity than OBP-301 by induction of p53-mediated apoptosis, in addition to oncolytic cell death [11, 12].

The protagonist in cancer immunotherapy is CD8 + T cells. Naive CD8 + T cells differentiate into antigen-specific effector T cells via T cell priming. The majority of these effector T cells, including the short-lived effector cells (SLECs), die via apoptosis after antigen clearance, and a small subset survives as long-lived memory T cells including the memory precursor effector cells (MPECs). MPECs further differentiate into tissue-resident memory T cells (TRMs) and effector memory T cells (TEMs) via the effector memory precursor cells (TEMps), and central memory T cells (TCMs) via the central memory precursor cells (TCMps) [13, 14]. Of these, TRMs in particular are reported to have an important role in long-term anti-tumor immunity in various cancers including pancreatic cancer [15–20]. IL-15Rα and extracellular adenosine triphosphate (ATP), well-known as an ICD marker [21], are reported to have important roles in the establishment, maintenance, and functionality of long-lived memory CD8 + T cell populations [22, 23].

In the present study, whether OBP-702 local treatment in combination with chemotherapy has the potential to increase systemic anti-tumor immunity in pancreatic cancer was investigated, focusing especially on the effects on the long-lived memory CD8 + T cells including MPECs, TEMs, and TRMs. Not only transient effects of short-lived effector CD8 + T cells, but also acquisition of long-term anti-tumor immunity via activation of long-lived memory CD8 + T cells would bring long-term sustained anti-tumor effects in pancreatic cancer.

## Methods

### TCGA analysis

The mRNA expression profiles and patient information of tumor samples were extracted from cBioPortal for Cancer Genomics (https://www.cbioportal.org/). The group determination of CD8 and CD103 expression was done based on median z-scores.

### Cell lines and cultures

The human pancreatic cancer cell line PANC-1, the human lung cancer cell line H1299, and the murine colon carcinoma cell line CT26 were purchased from the American Type Culture Collection (ATCC, Manassas, VA, USA), and the murine pancreatic cancer cell line PAN02 was purchased from the National Cancer Institute (Frederick, MD, USA). H1299, CT26, and PAN02 were cultured in RPMI1640 medium, and PANC-1 was cultured in DMEM medium. All culture media were supplemented with 10% fetal bovine serum and 1% penicillin-streptomycin (100 U/mL).

### Recombinant adenoviruses and chemotherapeutic agents

The structures of recombinant adenoviruses are shown in Supplementary Fig. S1. OBP-301 is a telomerase-specific oncolytic adenovirus, in which the human telomerase reverse transcriptase (hTERT) promoter drives the expression of the E1A and E1B genes. OBP-401 is an OBP-301 variant expressing green fluorescent protein (GFP). OBP-702 is another OBP-301 variant expressing p53 under control of the early growth response 1 (Egr1) promoter. Ad-p53 is a replication-deficient adenovirus expressing p53. Multiplicity of infection (MOI) and plaque-forming units (PFUs) were used as virus units in vitro and in vivo, respectively. GEM was purchased from Eli Lilly Japan K.K. (Kobe, Hyogo, Japan), and nab-PTX was purchased from Taiho (Tokyo, Japan).

### Western blot analysis

Proteins extracted from cells were electrophoresed on 10% SDS-polyacrylamide gels and transferred to Hybond-polyvinylidene difluoride membranes (GE Healthcare UK Ltd., Amersham, UK). The membranes were incubated overnight at 4 °C with primary antibodies against AIF (1:1000, 5318; Cell Signaling Technology, Danvers, MA, USA), E1A (1:1000, 554155; BD Pharmingen, San Jose, CA, USA), p53 (1:1000, 18032; Cell Signaling Technology), and β-actin (1:5000, A-5441; Sigma-Aldrich, St. Louis, MO, USA), followed by incubation with secondary antibodies for 1 h at room temperature. The Amersham ECL chemiluminescence system (GE Healthcare UK Ltd.) was used to detect peroxidase activity of the bound antibody.

### ATP assay

PANC-1, PAN02, and CT26 cells were treated with chemotherapeutic agents or viruses for 24 h, and levels of extracellular ATP were measured using an ENLITEN ATP assay (Promega, Madison, WI, USA).

### Flow cytometry

Lymphocytes from splenocytes were treated by red blood cell (RBC) lysis buffer (420302; BioLegend) and debris removal solution kit (Miltenyi Biotec, Bergisch, Gladbach, Germany). Tumor infiltrating lymphocytes (TILs) were treated by RBC lysis buffer and TTDR kit (661563; BD Biosciences). Lymphocytes or monocytes were treated by Fc receptor blocker (TruStain FcX PLUS, S17011E; BioLegend). After discrimination of live/dead cells by zombie aqua (423102; BioLegend) or zombie NIR (423106; BioLegend), cells were stained by PerCP CD3ε (100326; BioLegend), APC/Cy7 CD4 (100525; BioLegend), FITC CD8α (100706; BioLegend), FITC CD11b (101206; BioLegend), PerCP CD11c (117326; BioLegend), BV510 CD27 (124229; BioLegend), PE-Cy7 CD44 (103030; BioLegend), PE CD45 (103106; BioLegend), APC CD62L (104412; BioLegend), PE-Cy7 CD127 (135022; BioLegend), APC CD215(IL-15Rα) (153506; BioLegend), or BV421 KLRG1 (423102; BioLegend). All data were analyzed using FACS lyric or FACS ARIAIII (BD Biosciences) and FlowJo Software ver. 8.1 (BD Biosciences).

### In vivo experiments

PAN02 cells (2 × 10^6^ cells) subcutaneously injected into the flank of 6-week-old female C57BL/6 mice were treated with OBP-702 intratumoral injection (1.0 × 10^8^ PFUs or 5.0 × 10^7^ PFUs) and/or GN intraperitoneal injection (GEM 50 mg/kg, nab-PTX 5 mg/kg) (not randomized, not blinded, *n* = 3–7). A-438079 (80 mg/kg, A9736; Sigma-Aldrich), a P2RX7 purinergic receptor blocker, was used to block ATP [24]. Tumor volume was calculated using the following formula: tumor volume (mm^3^) = *a* × *b*^2^ × 0.5, where *a* represents the longest diameter, *b* represents the shortest diameter, and 0.5 is a constant used to calculate the volume of an ellipsoid. All animal experimental protocols were approved by the Institutional Animal Care and Use Committee of Okayama University.

### Immunohistochemical staining

Four-µm-thick tissue sections prepared from formalin-fixed and paraffin-embedded tissue samples were deparaffinized in xylene twice for 10 min each and rehydrated in a graded ethanol series for 10 min each. After blocking endogenous peroxidase by 3% hydrogen peroxide and antigen retrieval by 14 min of microwave heating in EDTA buffer (pH 7.4), the samples were incubated with primary antibodies against CD8 (1:400, ab199016, Abcam, Cambridge, UK), CD103 (1:200, ab129202, Abcam), and CD69 (1:200, bs-2499R, Bioss, Woburn, MA, USA) overnight at 4 °C, and then incubated with peroxidase-linked secondary antibody for 30 min at room temperature. The samples were immersed in 3, 3-diaminobenzidine (DAB) and counterstained with Mayer’s hematoxylin, followed by dehydration in a graded ethanol series for 10 min each. After mounting, the samples were subjected to microscopic observation. For fluorescence immunohistochemical staining for CD8 and CD103, Alexa 488 Anti-mouse (1:500, A11017, Invitrogen, Tokyo, Japan) or Alexa 647 Anti-Rabbit (1:500, A21245, Invitrogen) was used as the secondary antibody, and DAPI (D21490, FluoroPure™ grade, Invitrogen) was used for DNA staining.

### Clinical samples

Patients with borderline resectable pancreatic ductal adenocarcinoma (BR), who underwent surgical resection at Okayama University Hospital between 2012 and 2019, were divided into four groups, according to prognosis (good or poor) and treatment strategy (NAC or upfront surgery): good prognosis and NAC (G-N); good prognosis and upfront surgery (G-U); poor prognosis and NAC (P-N); and poor prognosis and upfront surgery (P-U). The cutoff period for a good or poor prognosis was defined as 24-month overall survival (OS). Three patients were selected in each group, and immunohistochemical staining for CD8 and CD103 of their surgical specimens was performed. This study, a retrospective study with an opt-out method, was approved by the institutional review board of Okayama University (No. 2110-017).

### Statistical analysis

Statistical analysis was performed using GraphPad Prism 9 software (San Diego, CA, USA). Student’s *t*-test, the Mann–Whitney *U* test, and one-way ANOVA were used for statistical analysis of continuous variables, with *P* values < 0.05 considered significant.

## Results

### Tissue-resident memory T cells have an essential role in the prognosis of pancreatic cancer

**Fig. 1:**
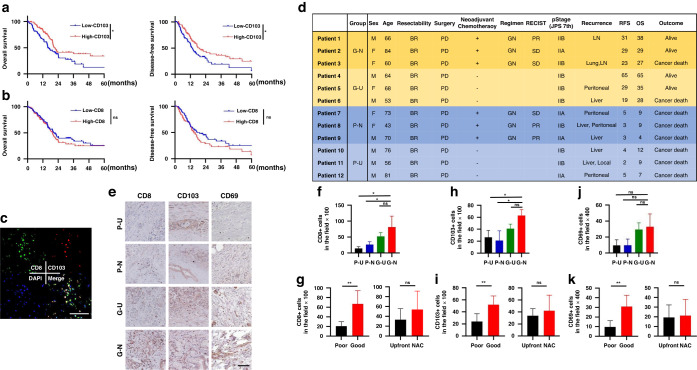
TCGA database analysis and CD8+ and CD103 + T cells in clinical specimens of pancreatic cancer. **a** Kaplan–Meier curves for overall survival and disease-free survival for high-CD103 and low-CD103 pancreatic cancers from the TCGA database (*n* = 187). **b** Kaplan–Meier curves for overall survival and disease-free survival for high-CD8 and low-CD8 pancreatic cancers (*n* = 187). **c** Representative figures of immunofluorescent staining for CD8 (green), CD103 (red), DAPI (blue), and merge, for which the surgical specimen of patient 1 was used. Note that colocalization (yellow) of CD8 and CD103 means TRMs. Scale bar, 100 µm. **d** Clinicopathological characteristics of 12 patients with borderline resectable pancreatic ductal adenocarcinoma who underwent surgical resection are shown. G-N, G-U, P-N, and P-U mean good prognosis and NAC, good prognosis and upfront surgery, poor prognosis and NAC, and poor prognosis and upfront surgery, respectively. The cutoff period for a good or poor prognosis was defined as 24-month OS. **e** Twelve surgical specimens were subjected to immunohistochemical staining for CD8, CD103, and CD69. Representative images of each group (G-N, G-U, P-N, and P-U) are shown in (**e**). Scale bar, 200 µm. **f**, **g** The number of CD8+ TILs assessed in six different, randomly selected fields is compared among 4 groups (*n* = 3) (**f**), and between poor and good prognosis groups and between upfront and NAC groups (*n* = 6) (**g**). **h**, **i** The number of CD103+ TILs assessed in six different, randomly selected fields is compared among 4 groups (*n* = 3) (**h**), and between poor and good prognosis groups and between upfront and NAC groups (*n* = 6) (**i**). **j**, **k** The number of CD69+ TILs assessed in six different, randomly selected fields is compared among four groups (*n* = 3) (**j**), and between poor and good prognosis groups and between upfront and NAC groups (*n* = 6) (**k**). *P < 0.05, **P < 0.005, ***P < 0.001. RFS relapse-free survival, OS overall survival, BR borderline resectable, PD pancreatoduodenectomy, GN gemcitabine + nab-paclitaxel, PR partial response, SD stable disease, NAC neoadjuvant chemotherapy.

When the clinical importance of TRMs was first checked in the TCGA public database, the expression of CD103 was significantly correlated with OS and disease-free survival, but CD8 was not (Fig. 1a, b). The clinical importance of TRMs was then analyzed using surgical specimens of pancreatic cancer resected after NAC in our hospital. Immunofluorescent staining showed that cells with colocalization of CD8 and CD103, meaning TRMs, were present in tumor tissues (Fig. 1c). Twelve pancreatic cancer patients were divided into four groups, three patients in each group, according to prognosis (good or poor) and treatment strategy (NAC or upfront surgery) (Fig. 1d). There were more CD8 +, CD103 +, and CD69+ cells in specimens of patients with a good prognosis, irrespective of treatment strategy (Fig. 1e–k).

### OBP-702 has cytotoxic effects on human and murine pancreatic cancer cell lines

Since OBP-702, which has the basic structure of human adenovirus type 5, infects cells via coxsackie adenovirus receptors (CAR) and integrin αvβ5 receptors [25], OBP-702 infected murine PAN02 and CT26 cells, which have integrin αvβ5 expression, but little CAR expression (Supplementary Fig. S2a), replicated after infection (Supplementary Fig. S2b), and produced cytotoxic effects as well as human PANC-1 cells (Supplementary Fig. S2c). OBP-401 infected and showed GFP on PANC-1 and PAN02 cells, but not on normal murine splenocytes (Supplementary Fig. S2d), which showed the tumor specificity of these oncolytic viruses. As for cytotoxic mechanisms, OBP-702 increased p53 expression and decreased p62 expression time-dependently (Supplementary Fig. S2e), and significantly increased active caspase-3 (Supplementary Fig. S2f, g), showing that OBP-702 produced cytotoxicity through apoptosis and autophagy in both human and murine cells.

### OBP-702 stimulates long-lived memory CD8 + T cells

**Fig. 2:**
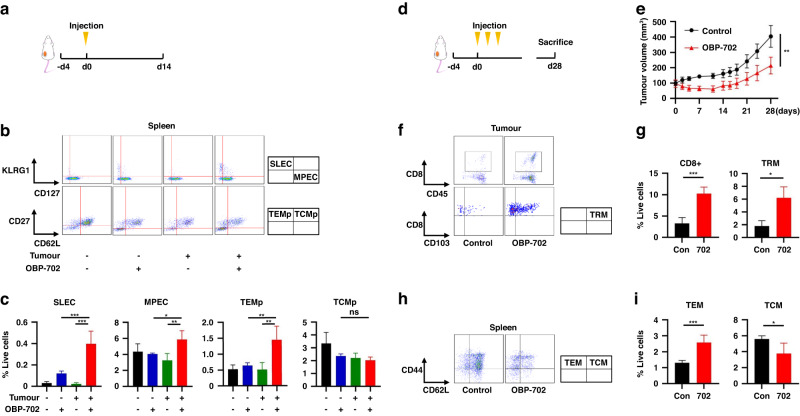
Activation of long-lived memory CD8 + T cells by OBP-702 in a PAN02 subcutaneous tumor model. **a**–**c** C57BL/6 mice bearing or not bearing PAN02 subcutaneous tumors were intratumorally treated with OBP-702 (1 × 10^8^ PFU) or PBS once and sacrificed 14 days after treatment (**a**). Splenocytes collected from the harvested spleen were subjected to flow cytometry for SLECs, MPECs, TEMps, and TCMps. Representative figures of flow cytometry are shown in (**b**), in which CD3+/CD8+ cells are analyzed in the upper figures, and CD3+/CD8+/CD127+ cells are analyzed in the lower figures. Populations of SLECs, MPECs, TEMps, and TCMps are compared among control, OBP-702 vaccination, tumor inoculation, and OBP-702 treatment to tumor (*n* = 3–4) (**c**). **d**, **e** C57BL/6 mice bearing PAN02 subcutaneous tumors were intratumorally treated with PBS or OBP-702 (5.0 × 10^7^ PFU) three times in a week and sacrificed 28 days after the initial treatment (**d**). Tumor volume was monitored after treatment by OBP-702 or PBS (*n* = 5) (**e**). **f**, **g** PAN02 tumors harvested at 28 days after the initial treatment with OBP-702 were subjected to flow cytometry for CD8+ cells and TRMs. Representative figures of flow cytometry are shown in (**f**), in which live cells are analyzed in the upper figures, and CD8+/CD45+ cells are analyzed in the lower figures. Populations of CD8+ cells and TRMs are compared between control and OBP-702 (*n* = 3–5) (**g**). **h**, **i** The spleens harvested 28 days after the initial treatment with OBP-702 were subjected to flow cytometry for TEMs and TCMs. Representative figures of flow cytometry are shown in (**h**), in which CD3+/CD8+ cells are analyzed. Populations of TEMs and TCMs are compared between control and OBP-702 (*n* = 5) (**i**). *P < 0.05, **P < 0.005, ***P < 0.001. SLEC short-lived effector T cell, MPEC memory precursor effector cell, TEMp effector memory precursor cell, TCMp central memory precursor cell, TRM tissue-resident memory T cell, TEM effector memory T cell, TCM central memory T cell, Con control, 702 OBP-702.

When effects of OBP-702 treatment to PAN02 tumors on long-term anti-tumor immunity were first examined in a comparison of four groups (control, OBP-702 vaccination, tumor inoculation, and OBP-702 treatment to tumor), OBP-702 treatment induced more SLECs, MPECs, and TEMps in the spleen (Fig. 2a–c). Local OBP-702 treatment showed significantly stronger anti-tumor effects on PAN02 tumors (Fig. 2d, e) and increased CD8 + T cells and TRMs in the tumor and TEMs in the spleen 28 days after the initial treatment (Fig. 2f–i). Similar effects were observed on CT26 tumors as well (Supplementary Fig. S3).

### OBP-702 promotes the expressions of ATP and IL-15Ra, which stimulate long-term immunity

**Fig. 3:**
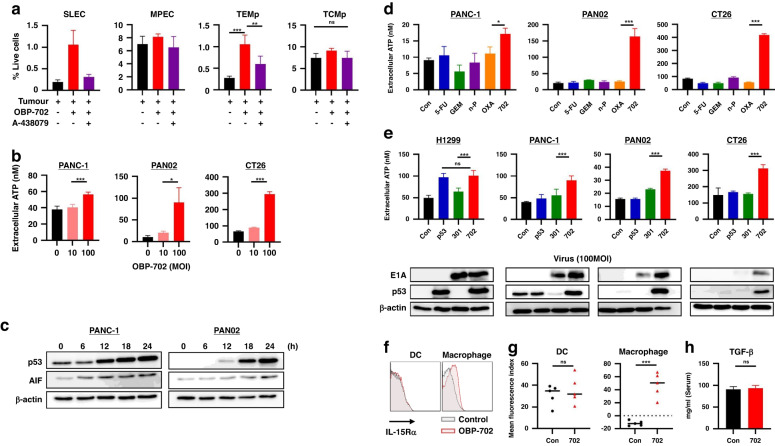
Stimulation of long-term immunity by OBP-702 via virus infection and p53 production. **a** A-438079 was intraperitoneally injected into mice 6 h before OBP-702 treatment to PAN02 tumors that had been treated for 6 days to block P2RX7 receptors, and populations of SLECs, MPECs, TEMps, and TCMps in the splenocytes were analyzed by flow cytometry 7 days after treatment (*n* = 3–5). **b** Extracellular ATP secreted from PANC-1, PAN02, and CT26 cells treated with OBP-702 (0, 10, and 100 MOI) was measured with a luminescence assay 24 h after treatment (*n* = 3–5). **c** Whole cell lysates of PANC-1 and PAN02 cells collected at the indicated time points (0, 6, 12, 18, 24 h) after OBP-702 treatment (100 MOI) were subjected to western blotting for p53, AIF, and β-actin. **d** Extracellular ATP secreted from PANC-1, PAN02, and CT26 cells after monotherapy with chemotherapeutic agents (5-FU, GEM, nab-PTX, and OXA) and OBP-702 at the IC50 dose was measured with a luminescence assay 24 h after treatment (*n* = 3). **e** Extracellular ATP secreted from H1299, PANC-1, PAN02, and CT26 cells after treatment with Ad-p53, OBP-301, or OBP-702 at 100 MOI was measured with a luminescence assay 24 h after treatment (*n* = 3–5). Western blots of p53 and E1A are shown corresponding to the upper graph. **f**, **g** The spleens harvested 9 days after the initial treatment with OBP-702 were subjected to flow cytometry for IL-15-Rα on the surface of DCs (CD11b+/CD11c+/MHC-Class II+) and macrophages (CD11b+ except DC). Representative figures of flow cytometry are shown in (**f**), and the mean expression levels of IL-15-Rα are compared between control and OBP-702 (*n* = 5) (**g**). **h** The ELISA data show the murine serum TGF-β1 levels collected from the mice treated with PBS or OBP-702 7 days after treatment (*n* = 5). *P < 0.05, **P < 0.005, ***P < 0.001. SLEC short-lived effector T cell, MPEC memory precursor effector cell, TEMp effector memory precursor cell, TCMp central memory precursor cell, ATP adenosine triphosphate, MOI multiplicity of infection, AIF apoptosis-inducing factor, Con control, 5-FU 5-fluorouracil, GEM gemcitabine, n-P nab-paclitaxel, OXA oxaliplatin, 702 OBP-702, 301 OBP-301, DC dendritic cell.

A-438079, the P2RX7 antagonist, significantly blocked induction of SLECs and TEMps, but not MPECs (Fig. 3a), which suggested that ATP was involved in induction of SLECs and TEMps. It has been reported that p53 is involved in ATP induction via activation of the apoptosis-inducing factor (AIF) [26]. OBP-702 actually induced extracellular ATP dose-dependently through time-dependent AIF activation associated with p53 upregulation (Fig. 3b, c). When ATP induction by OBP-702 was compared with representative chemotherapeutic agents such as 5-FU, GEM, nab-PTX, and oxaliplatin (OXA) at the half maximal inhibitory concentration (IC50) dose (Supplementary Fig. S4a), OBP-702 induced significantly more ATP than the other chemotherapeutic agents (Fig. 3d).

Cytotoxic mechanisms of OBP-702 are roughly divided into p53 induction and viral replication. ATP levels in supernatants of H1299, PANC-1, and PAN02 cells were significantly correlated with p53 expression (Supplementary Fig. S4b) and viral replication shown by adenoviral E1A levels (Supplementary Fig. S4c), but no ATP increase was observed on CT26 cells in which p53 was not induced and little E1A expression was observed. In fact, OBP-702 induced ATP more strongly than the other viruses on all cell lines in which E1A and p53 expressions were observed after OBP-702 treatment (Fig. 3e). Although there would be one possibility that intracellular ATP was just released into supernatant after cell death caused by these virus treatments, 100 MOI of these viruses did not always show cytotoxic effects 24 h after treatment (Supplementary Fig. S4d).

TGF-β and IL-15 are reported to play key roles in the control of effector CD8 + T cells by exerting opposing effects [27], and the complex of IL-15 with IL-15Rα, IL-15/IL-15Rα, results in persistent activity and prolonged survival of memory CD8 + T cells [28]. The origin of IL-15Rα, macrophages or dendritic cells, divides CD8 + T cell subsets [22]. OBP-702 treatment increased IL-15Rα-expressing macrophages in the spleen, but not IL-15Rα-expressing DCs (Fig. 3f, g), whereas no change was observed in serum TGF-β expression (Fig. 3h).

### GN plus OBP-702 combination therapy shows strong synergistic and sustained anti-tumor effects on pancreatic cancer

When the combination effects of OBP-702 with GN were first investigated in vitro, GN + OBP-702 showed significantly strong and synergistic cytotoxic effects (Supplementary Fig. S5a, b). OBP-702 induced E1A and p53 expressions in combination with GN (Supplementary Fig. S5c). Whereas OBP-702 monotherapy increased the multinuclear (MN) phase in the cell cycle, GN + OBP-702 significantly increased the MN phase compared to GN (Supplementary Fig. S5d–g), and induced apoptosis more strongly than GN or OBP-702 monotherapy (Supplementary Fig. S5h, i). Although GN did not affect ATP production, the addition of OBP-702 induced dose-dependent ATP production (Supplementary Fig. S5j, k).

**Fig. 4:**
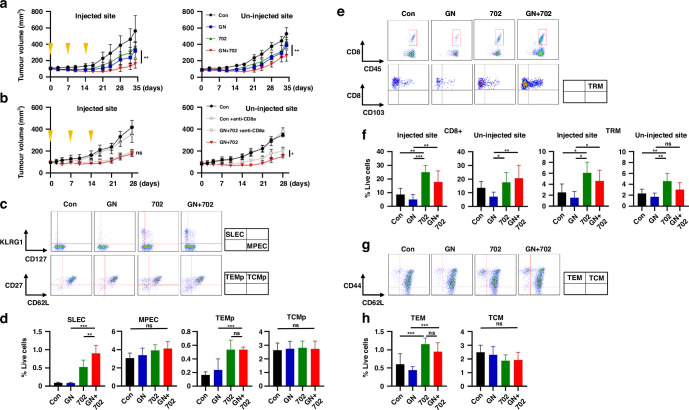
Abscopal effect of combination therapy of GEM and nab-PTX with OBP-702 in a PAN02 bilateral subcutaneous tumor model. **a** C57BL/6 mice bilaterally bearing PAN02 subcutaneous tumors were treated with GN (GEM 50 mg/kg, nab-PTX 5 mg/kg) intraperitoneally and/or OBP-702 (5 × 10^7^ PFU) intratumorally once a week for 3 weeks, and sacrificed 34 days after initial treatment. OBP-702 was injected into the bigger tumor. Tumor volume was monitored (*n* = 7). The left figure shows tumor volume of the OBP-702-injected side, and the right figure shows the tumor volume of the OBP-702-uninjected side. **b** Anti-CD8α antibody was additionally injected into the peritoneal cavity once a week for 4 weeks, and tumor volume of control and OBP-702 with or without anti-CD8α antibody was monitored until 28 days after the initial treatment (*n* = 3–7). **c**, **d** The spleens harvested 14 days after the initial treatment were subjected to flow cytometry for SLECs, MPECs, TEMps, and TCMps. Representative figures of flow cytometry are shown in (**c**), in which CD3+/CD8+ cells are analyzed in the upper figures, and CD3+/CD8+/CD127+ cells are analyzed in the lower figures. Populations of SLECs, MPECs, TEMps, and TCMps are compared (*n* = 5) (**d**). **e**, **f** PAN02 tumors harvested 34 days after the initial treatment were subjected to flow cytometry for CD8+ cells and TRMs. Representative figures of flow cytometry are shown in (**e**), in which live cells are analyzed in the upper figures, and CD8+/CD45+ cells are analyzed in the lower figures. Populations of CD8+ cells and TRMs in OBP-702-injected tumors and OBP-702-uninjected tumors are compared (*n* = 3–7) (**f**). **g**, **h** The spleens harvested 34 days after the initial treatment were subjected to flow cytometry for TEMs and TCMs. Representative figures of flow cytometry are shown in (**g**), in which CD3+/CD8+ cells are analyzed. Populations of TEMs and TCMs are compared (*n* = 7) (**h**). *P < 0.05, **P < 0.005, ***P < 0.001. Con control, GN gemcitabine + nab-paclitaxel, 702 OBP-702, SLEC short-lived effector T cell, MPEC memory precursor effector cell, TEMp effector memory precursor cell, TCMp central memory precursor cell, TRM tissue-resident memory T cell, TEM effector memory T cell, TCM central memory T cell.

Next, in PAN02 bilateral subcutaneous tumor models, GN + OBP-702 significantly suppressed the growth of not only OBP-702-injected tumors, but also OBP-702-uninjected tumors (Fig. 4a), and this anti-tumor activity on uninjected tumors was significantly reduced by anti-CD8α injection (Fig. 4b and Supplementary Fig. S6a–c). On further analysis of memory T cells, OBP-702 and GN + OBP-702 significantly increased SLECs and TEMps 14 days after the initial treatment (Fig. 4c, d). OBP-702 and GN + OBP-702 increased CD8 + T cells and TRMs in injected and uninjected tumors (Fig. 4e, f) and increased TEMs in the spleen 34 days after the initial treatment (Fig. 4g, h). These results showed that GN + OBP-702 not only produced the abscopal effect by temporarily activating anti-tumor immunity via tumor specific CD8 + T cells, but it also stimulated long-term anti-tumor immunity by increasing TRMs and TEMs, which potentially results in sustained anti-tumor activity.

### Neoadjuvant GN + OBP-702 therapy suppresses the growth of re-inoculated tumors via activation of long-term immunity

**Fig. 5:**
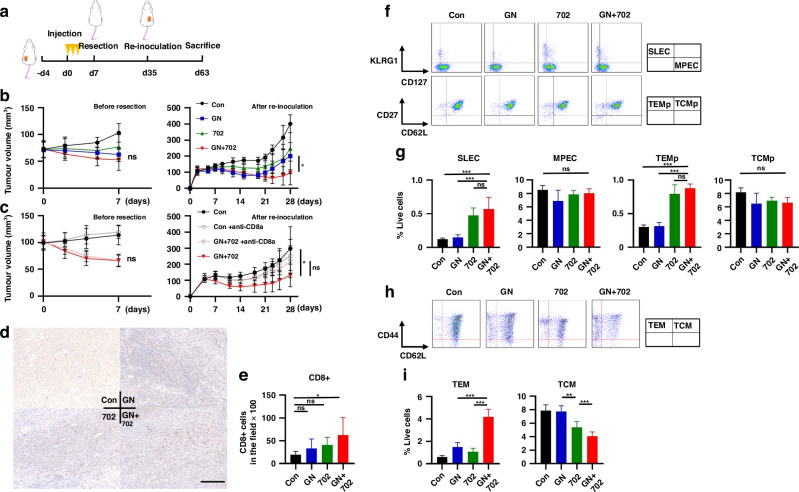
Activation of long-term anti-tumor immunity by GN + OBP-702 in a neoadjuvant model using PAN02 subcutaneous tumors. **a** Study protocol of a neoadjuvant model. C57BL/6 mice bearing PAN02 subcutaneous tumors were treated with GN (GEM 50 mg/kg, nab-PTX 5 mg/kg) intraperitoneally and/or OBP-702 (5 × 10^7^ PFU) intratumorally three times in a week, and these tumors were surgically resected 7 days after the initial treatment. PAN02 cells were re-inoculated into the opposite side of the flank to the first tumor 28 days after tumor resection, and mice were monitored with no treatment and sacrificed 28 days after re-inoculation. **b** Tumor volume was monitored for 7 days until resection (left) (*n* = 6–7) and for 28 days after re-inoculation with no treatment (right). **c** Anti-CD8α antibody was added once on the day of the initial treatment in the protocol (**a**). Tumor volume was monitored the same as in (**b**) (*n* = 7). **d**, **e** PAN02 tumors harvested 28 days after re-inoculation were subjected to immunohistochemical staining for CD8. Representative images of immunohistochemical staining are shown in (**d**). Scale bar, 200 µm. The number of CD8+ TILs, which were evaluated in three different, randomly selected fields, were compared (*n* = 5-7) (**e**). **f**, **g** The spleens harvested 14 days after the initial treatment in the protocol (**a**) were subjected to flow cytometry for SLECs, MPECs, TEMps, and TCMps. Representative figures of flow cytometry are shown in (**f**), in which CD3+/CD8+ cells are analyzed in the upper figures, and CD3+/CD8+/CD127+ cells are analyzed in the lower figures. Populations of SLECs, MPECs, TEMps, and TCMps are compared (*n* = 5) (**g**). **h**, **i** The spleens harvested 35 days after the initial treatment in the protocol (**a**) were subjected to flow cytometry for TEMs and TCMs. Representative figures of flow cytometry are shown in (**h**), in which CD3+/CD8+ cells are analyzed. Populations of TEMs and TCMs are compared (*n* = 5) (**i**). *P < 0.05, **P < 0.005, ***P < 0.001. Con control, GN gemcitabine + nab-paclitaxel, 702 OBP-702, SLEC short-lived effector T cell, MPEC memory precursor effector cell, TEMp effector memory precursor cell, TCMp central memory precursor cell, TEM effector memory T cell, TCM central memory T cell.

A re-challenge model to imitate NAC in clinical practice was designed (Fig. 5a). The growth of re-inoculated tumors was significantly suppressed in mice treated with GN + OBP-702 as NAC (Fig. 5b), and no specific side effects were observed based on body weight change (Supplementary Fig. S7a) and histological findings of major organs (Supplementary Fig. S7b). After confirmation of deletion of CD8 + T cells by anti-CD8α antibody being continued for 1 to 2 weeks (Supplementary Fig. S6a–c), when mice were injected with anti-CD8α antibody at initial treatment, the significant anti-tumor effect observed in mice treated with GN + OBP-702 disappeared (Fig. 5c). More CD8 + T cells were observed in re-inoculated tumors of mice treated with GN + OBP-702 (Fig. 5d, e). SLECs and TEMps in the spleen were significantly increased 14 days after the initial treatment, when the tumors had already been surgically resected, in mice treated with OBP-702 and GN + OBP-702 (Fig. 5f, g). TEMs in the spleen were also significantly increased in mice treated with GN + OBP-702 35 days after the initial treatment (at the timing of re-inoculation) (Fig. 5h, i).

## Discussion

Oncolytic virotherapy (OV) is an emerging treatment strategy that will be less invasive than surgery, with aspects of both local therapy via potent direct cytotoxic effects and systemic therapy via activation of systemic anti-tumor immunity. These notable characteristics are not found in the current treatment strategies such as surgery, chemotherapy, and immunotherapy. Various oncolytic viruses have been developed worldwide [29], and Talimogene laherparepvec (T-VEC), a genetically modified herpes simplex virus expressing GM-CSF, is at the frontier of OV. The phase III clinical study of a single injection of T-VEC for melanoma (OPTiM Study) showed that the complete response (CR) rate was 10.8%, and the phase Ib clinical study of T-VEC combined with pembrolizumab (Masterkey-265 Study) showed that the CR rate was 43% [30]. Several clinical studies to investigate the efficacy of OV in the NAC setting are currently ongoing [31]. We previously developed OBP-301 and investigated the combination effects with other treatment modalities such as chemotherapy and radiotherapy [7, 32, 33]. After the phase I clinical study to determine clinical safety in the US [34], we conducted the phase I clinical study in Japan to examine the feasibility of OBP-301 combined with radiotherapy for esophageal cancer, in which the CR rate of this combination was as high as 66.7% [8]. OBP-301 is now being tested in the phase II clinical study to verify the efficacy of OBP-301 with radiotherapy for esophageal cancer in Japan, and it is also being tested in several other clinical studies of its combination with ICIs in Japan and other countries.

In the present study, OBP-702, an OBP-301 variant with p53 gene, which can produce potent therapeutic effects on aggressive cancers such as pancreatic cancer, on which OBP-301 shows little effect, was used [11]. One of the main characteristics of OBP-702 is the presence of the p53 tumor suppressor gene. p53, known as “the guardian of the genome”, regulates cell division and prevents tumor formation by controlling the cell cycle and DNA repair machinery, and p53 replacement therapy has shown potent anti-tumor effects in preclinical and clinical studies [35, 36]. In addition, p53 is known to have a fundamental role in tumor metabolism and to stimulate mitochondrial oxidative phosphorylation via AIF, GLS2, and SCO2, which activates the induction of ATP [26]. In the present study, OBP-702 induced ATP more strongly than 5-FU, GEM, nab-PTX, and OXA, and OBP-301, which suggested that OBP-702 was an oncolytic adenovirus possessing a high potential to strongly activate anti-tumor immunity, and p53 played an important role in that mechanism. As for the effect of p53 on anti-tumor immunity, there is a report that local activation of p53 by nutlin-3a enhanced anti-tumor immunity by overcoming immune suppression in the tumor microenvironment [37], which suggests that OBP-702 possibly possesses a similar function. Actually, we have previously shown the potential of OBP-702 to overcome immune suppression by inducing apoptosis and autophagy on cancer-associated fibroblasts via p53 overexpression [38]; however, its effects on long-term anti-tumor immunity are still unclear. OBP-702 may be also useful as a carcinopreventive agent for pre-malignant lesions such as intraductal papillary mucinous neoplasm (IPMN), of which malignant transformation was reportedly strongly associated with hTERT expression [39]. This will be an important point in drug development for pancreatic cancer, in which the tumor microenvironment is a key barrier to suppressing anti-tumor immunity and limiting the efficacy of immunotherapy [4, 40], and OBP-702 will need further investigation of this matter. For clinical application, the pharmacokinetic and toxicological assessments of OBP-702 have been comprehensively conducted at the Southern Research Institute (Alabama, USA) and the BoZo Research Centre (Tokyo, Japan), and have confirmed the safety of OBP-702 (unpublished data).

Whereas OV has been well recognized as a strong activator of anti-tumor immunity and produces an abscopal effect on metastatic lesions, mainly brought about by temporal effector CD8 + T cells of SLECs, little is known about the effect of OV on long-term anti-tumor immunity, as with conventional chemotherapy and radiotherapy. Given this background, the focus in the present study was more on the influence on long-term immunity rather than short-term immunity, and it showed that OBP-702 increased TRMs and TEMs via TEMp induction. TRMs are considered an especially important subset for long-term immunity. CD103, a receptor for E-cadherin, and CD69, an inhibitor of the sphingosine 1-phosphate receptor (S1P1), are both involved in the cellular migration process and recognized as representative TRM markers [41, 42]. High TRM population was actually found to be a factor associated with a good prognosis in the analysis using surgical specimens of pancreatic cancer. With respect to mechanisms for regulation of long-term anti-tumor immunity, ATP was reportedly involved in the establishment and maintenance of memory CD8 + T cells [23], and blocking ATP by a purinergic receptor P2RX7 blocker, A-438079, was shown to inhibit the induction of SLECs and TEMps by OBP-702. Another factor involved in the establishment of long-term immunity is the IL-15/ IL-15Rα complex [28]. Physiological functions are reported to differ depending on the cells expressing IL-15Rα, and IL-15Rα expressed on macrophages facilitates the transition of antigen-specific effector CD8 + T cells to memory cells and supports both TCMs and TEMs after differentiation to memory cells, whereas IL-15Rα expressed on DCs selectively supports TCMs [22]. OBP-702 significantly increased IL-15Rα on macrophages, but not on DCs.

In the present study, OBP-702 local treatment was proven to have the potential to produce strong and sustained anti-tumor activity by direct cytotoxicity and indirect systemic activation of anti-tumor immunity in the short-term and long-term on immunologically cold pancreatic cancer in combination with GN, which indicated that OBP-702 would be better used in the early phase of treatment including NAC. Although further investigation is needed, OBP-702 can be a promising treatment option for pancreatic cancer as an immunostimulant that can convert the tumor microenvironment immunologically from cold to hot following strong direct cytotoxic activity and provide durable anti-tumor effects through activation of long-lived memory CD8 + T cells, leading to a sustained survival benefit for pancreatic cancer patients.

## Supplementary information

Supplementary Figure

## Source data

Source Data

## Data Availability

The TCGA data shown in Fig. 1a, b were obtained from cBioPortal for Cancer Genomics (https://www.cbioportal.org/). All other data of this study are available in a source data file.
