# Author Correction: Long-term activation of anti-tumor immunity in pancreatic cancer by a p53-expressing telomerase-specific oncolytic adenovirus

**DOI:** 10.1038/s41416-024-02850-0

**Published:** 2024-10-14

**Authors:** Masashi Hashimoto, Shinji Kuroda, Nobuhiko Kanaya, Daisuke Kadowaki, Yusuke Yoshida, Masaki Sakamoto, Yuki Hamada, Ryoma Sugimoto, Chiaki Yagi, Tomoko Ohtani, Kento Kumon, Yoshihiko Kakiuchi, Kazuya Yasui, Satoru Kikuchi, Ryuichi Yoshida, Hiroshi Tazawa, Shunsuke Kagawa, Takahito Yagi, Yasuo Urata, Toshiyoshi Fujiwara

**Affiliations:** 1 Department of Gastroenterological Surgery, Okayama University Graduate School of Medicine, Dentistry and Pharmaceutical Sciences, Okayama, Japan; 2 Minimally Invasive Therapy Center, Okayama University Hospital, Okayama, Japan; 3 Center for Innovative Clinical Medicine, Okayama University Hospital, Okayama, Japan; 4 Clinical Cancer Center, Okayama University Hospital, Okayama, Japan; 5 Oncolys BioPharma, Inc., Tokyo, Japan

**Keywords:** Cancer immunotherapy, Immunotherapy

Correction to: *British Journal of Cancer* 10.1038/s41416-024-02583-0, published online 5 February 2024

In Fig. 4b, Uninjected site, the labels for ‘GN+702’ and ‘GN+702 +anti-CD8α’ were incorrectly placed; the labels should be transposed, as shown in the corrected figure presented here.
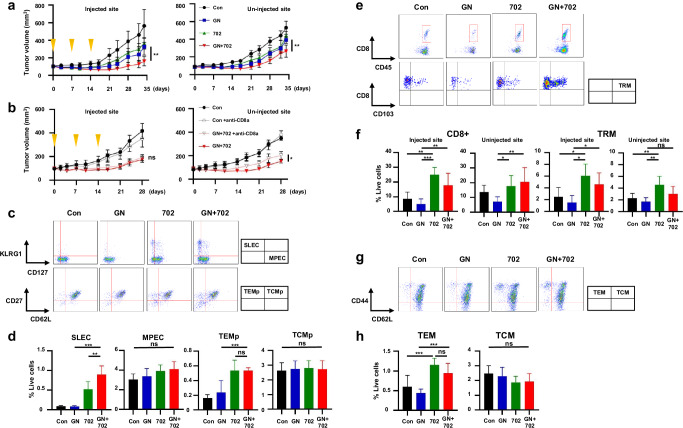


In Fig. 5c, After re-inoculation, the labels for ‘GN+702’ and ‘GN+702 +anti-CD8α’ were incorrectly placed; the labels should be transposed, as shown in the corrected figure presented here.
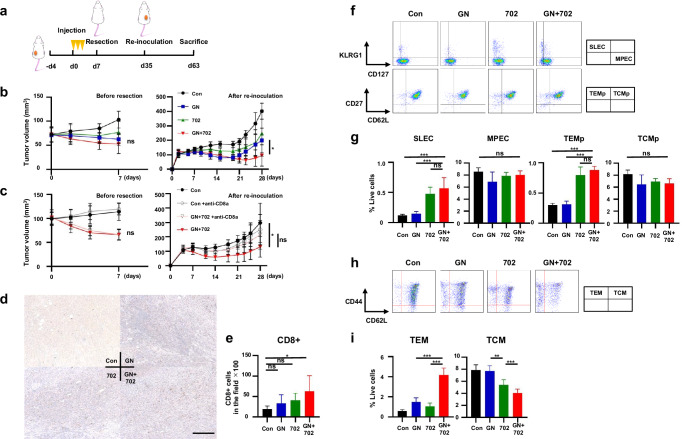


The correction does not have any effect on the results or conclusions of the paper.

The original article has been corrected.

