# Supplementary material for: Long-term activation of anti-tumor immunity in pancreatic cancer by a p53-expressing telomerase-specific oncolytic adenovirus
Source: Br J Cancer. 2024 Feb 5;130(7):1187–95. doi: 10.1038/s41416-024-02583-0 (PMC10991504; doi:10.1038/s41416-024-02583-0)
Supplement: Supplementary file 1 — Supplementary Figure [file 41416_2024_2583_MOESM1_ESM.pdf]

Supplementary Information

Long-term activation of anti-tumor immunity in pancreatic cancer by a p53-expressing telomerase-specific oncolytic adenovirus

Masashi Hashimoto[1], Shinji Kuroda[1,2]*, Nobuhiko Kanaya[1], Daisuke Kadowaki[1], Yusuke Yoshida[1], Masaki Sakamoto[1], Yuki Hamada[1], Ryoma Sugimoto[1], Chiaki Yagi[1], Tomoko Otani[1], Kento Kumon[1], Yoshihiko Kakiuchi[1,2], Kazuya Yasui[1], Satoru Kikuchi[1], Ryuichi Yoshida[1], Hiroshi Tazawa[1,3], Shunsuke Kagawa[1,4], Takahito Yagi[1], Yasuo Urata[5], Toshiyoshi Fujiwara[1]

[1] Department of Gastroenterological Surgery, Okayama University Graduate School of Medicine, Dentistry and Pharmaceutical Sciences, Okayama, Japan.
[2] Minimally Invasive Therapy Center, Okayama University Hospital, Okayama, Japan.
[3] Center for Innovative Clinical Medicine, Okayama University Hospital, Okayama, Japan.
[4] Clinical Cancer Center, Okayama University Hospital, Okayama, Japan.
[5] Oncolys BioPharma, Inc., Tokyo, Japan

Fig. S1.
Structures of recombinant adenoviruses (OBP-301, OBP-401, OBP-702, Ad-p53)

Fig. S2.
Infection and replication potential of OBP-702 in murine cell lines

Fig. S3.
Activation of long-lived memory CD8+ T cells by OBP-702 in a CT26 subcutaneous tumor model

Fig. S4.
Cytotoxic activity of recombinant adenoviruses and chemotherapeutic agents

Fig. S5.
Synergistic cytotoxic effects of OBP-702 with GEM and nab-PTX on human and murine pancreatic cancer cell lines

Fig. S6.
Supporting data for Fig. 4 and Fig. 5

Fig. S7.
Supporting data for Fig. 5

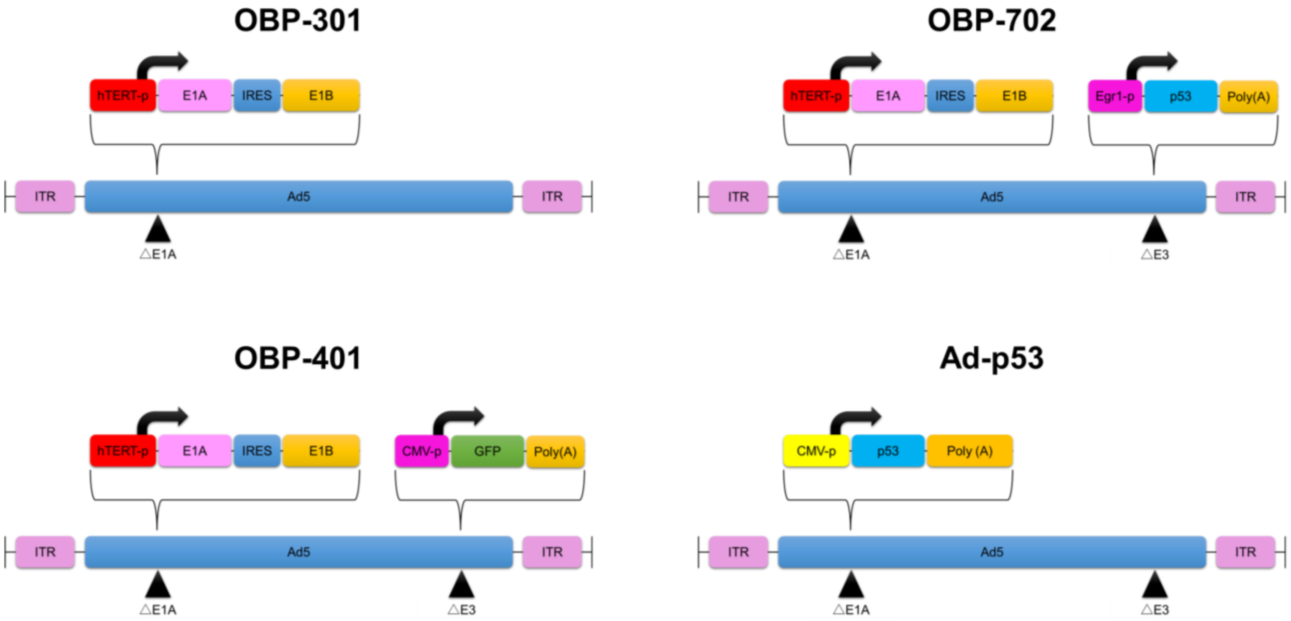

Fig. S1. Structures of recombinant adenoviruses (OBP-301, OBP-401, OBP-702, Ad-p53)

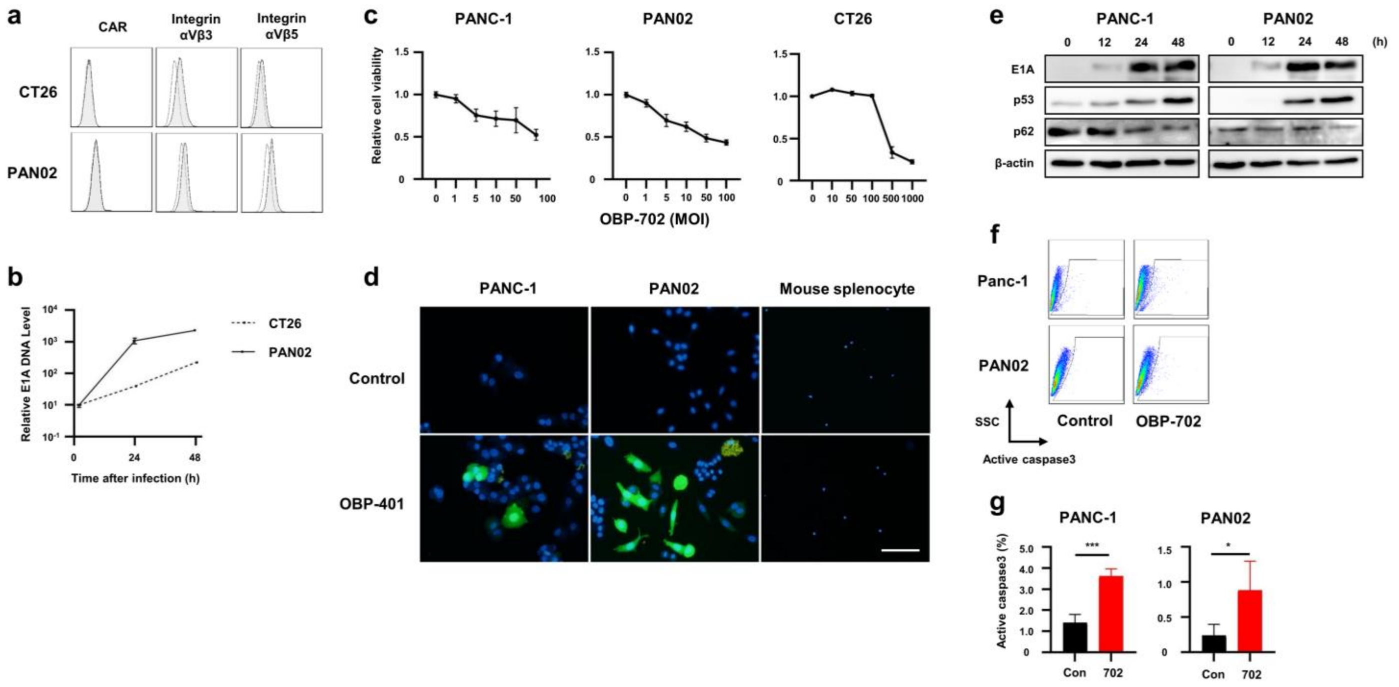

Fig. S2. Infection and replication potential of OBP-702 in murine cell lines

a. Expressions of coxsackie adenovirus receptors (CAR), Integrin αVβ3, and Integrin αVβ5 on the surfaces of the murine colon carcinoma cell line CT26 and the murine pancreatic cancer cell line PAN02 cells were analyzed by flow cytometry. **b.** CT26 and PAN02 cells treated with OBP-702 (10 MOI for PAN02, 100 MOI for CT26) were collected 2, 24, 48, and 72 hours after treatment and subjected to quantitative polymerase chain reaction (qPCR) after total DNA extraction using QIAmp DNA Mini Kit (Qiagen, Valencia, CA, USA). PCR amplification was conducted with 40 cycles of denaturation at 95 °C for 20 sec, with annealing at 60 °C for 20 sec. Data analysis was performed using a StepOnePlusTM real-time PCR system (Applied Biosystems, Waltham, MA, USA). Primers and probes were predesigned by the manufacturer (Applied Biosystems). TaqMan E1A probe: 5’-CTGTGTCTAGAGAATGC-MGB-3’, TaqMan E1A primers: E1A-F 5’-CCTGAGACGCCCGACATC-3’, E1A-R 5’-GGACCGGAGTCACAGCTATCC-3’. **c.** Viability of PANC-1, PAN02, and CT26 cells was assessed using an XTT assay 3 days after OBP-702 treatment at the indicated doses (MOI) (n=5). The percentage of viable cells relative to non-treated cells (0 MOI) is plotted. Error bars indicate 95% confidence intervals. **d.** Fluorescent images of PANC-1, PAN02, and mouse splenocytes were taken 24 hours after OBP-401 infection (100 MOI). Scale bar, 400 µm. **e.** Whole cell lysates of PANC-1 and PAN02 cells collected at the indicated time points (0, 12, 24, 48 hours) after OBP-702 treatment (10 MOI) were subjected to western blot analysis of E1A, p53, p62, and β-actin. **f,g.** PANC-1 and PAN02 cells were subjected to flow cytometry of active caspase-3 at 3 days after PBS (control) or OBP-702 treatment (10 MOI). Representative figures of flow cytometry are shown in (f), and the mean value of active caspase-3 is compared between control and OBP-702 (n=4) (g). *p <0.05, ***p <0.001. CAR, coxsackie adenovirus receptors; MOI, multiplicity of infection; Con, control; 702, OBP-702.

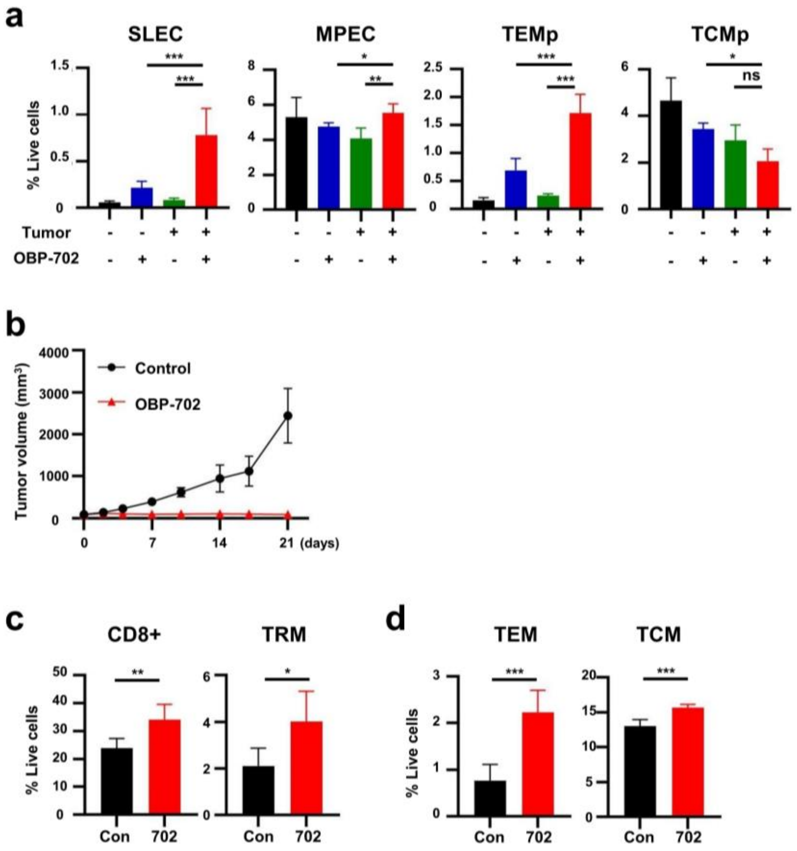

Fig. S3. Activation of long-lived memory CD8+ T cells by OBP-702 in a CT26 subcutaneous tumor model

a. BALB/c mice bearing or not bearing CT26 subcutaneous tumors were intratumorally treated with PBS or OBP-702 (1×10^{8} PFU) once and sacrificed 7 days after treatment. Splenocytes collected from the harvested spleen were subjected to flow cytometry for long-lived memory T cells (SLECs, MPECs, TEMps, and TCMps). Populations of SLECs, MPECs, TEMps, and TCMps are compared among control, OBP-702 vaccination, tumor inoculation, and OBP-702 treatment to tumor (n=5). **b.** BALB/c mice bearing CT26 subcutaneous tumors were intratumorally treated with PBS or OBP-702 (1.0×10^{8} PFU) 3 times in a week and sacrificed 21 days after the initial treatment. Tumor volume was monitored until 21 days after the initial treatment (n=6). **c.** CT26 tumors harvested 21 days after the initial treatment were subjected to flow cytometry for CD8+ cells and TRMs. Populations of CD8+ cells and TRMs are compared between control and OBP-702 (n=4-6). **d.** The spleens harvested at 21 days after the initial treatment were subjected to flow cytometry for TCMs and TEMs. Populations of TEMs and TCMs are compared between control and OBP-702 (n=6). *p <0.05, **p <0.005, ***p <0.001. SLEC, short-lived effector T cell; MPEC, memory precursor effector cell; TEMp, effector memory precursor cell; TCMp, central memory precursor cell; TRM, tissue-resident memory T cell; TEM, effector memory T cell; TCM, central memory T cell; Con, control; 702, OBP-702.

a

	OBP-702 (MOI)	5-FU (μM)	GEM (μM)	nab-PTX (nM)	OXA (μM)
PANC-1	81.3	1.7	0.9	9.4	13.1
PAN02	53.1	0.2	0.3	31.7	0.4
CT26	335.4	1.4	0.2	265.7	1.2

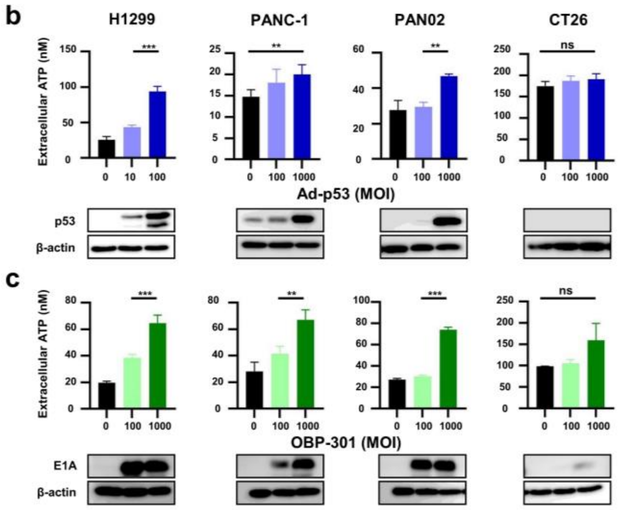

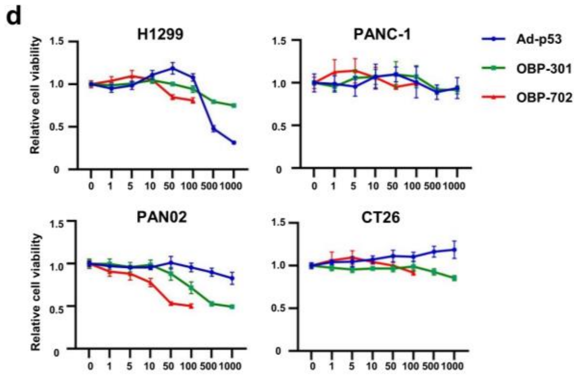

Fig. S4. Cytotoxic activity of recombinant adenoviruses and chemotherapeutic agents
a. The half maximal inhibitory concentration (IC50) dose of OBP-702 and chemotherapeutic agents (5-FU, GEM, nab-PTX, OXA) 3 days after treatment for PANC-1, PAN02, and CT26 cells. **b.** Extracellular levels of ATP secreted from H1299, PANC-1, PAN02, and CT26 cells after treatment with Ad-p53 at the indicated doses were measured with a luminescence assay 24 hours after treatment (n=4). Western blots of p53 are shown corresponding to the upper graph. **c.** Extracellular levels of ATP secreted from H1299, PANC-1, PAN02, and CT26 cells after treatment with OBP-301 at the indicated doses were measured with a luminescence assay 24 hours after treatment (n=3-4). Western blots of E1A are shown corresponding to the upper graph. **d.** Viability of the human lung cancer cell line H1299, PANC-1, PAN02, and CT26 cells was assessed using an XTT assay 24 hours after treatment with Ad-p53, OBP-301, or OBP-702 at the indicated doses (MOI) (n=5). The percentage of viable cells relative to non-treated cells (0 MOI) is plotted. Error bars indicate 95% confidence intervals. 5-FU, 5-fluorouracil; GEM, gemcitabine; nab-PTX, nab-paclitaxel; OXA, oxaliplatin; ATP, adenosine triphosphate; MOI, multiplicity of infection.

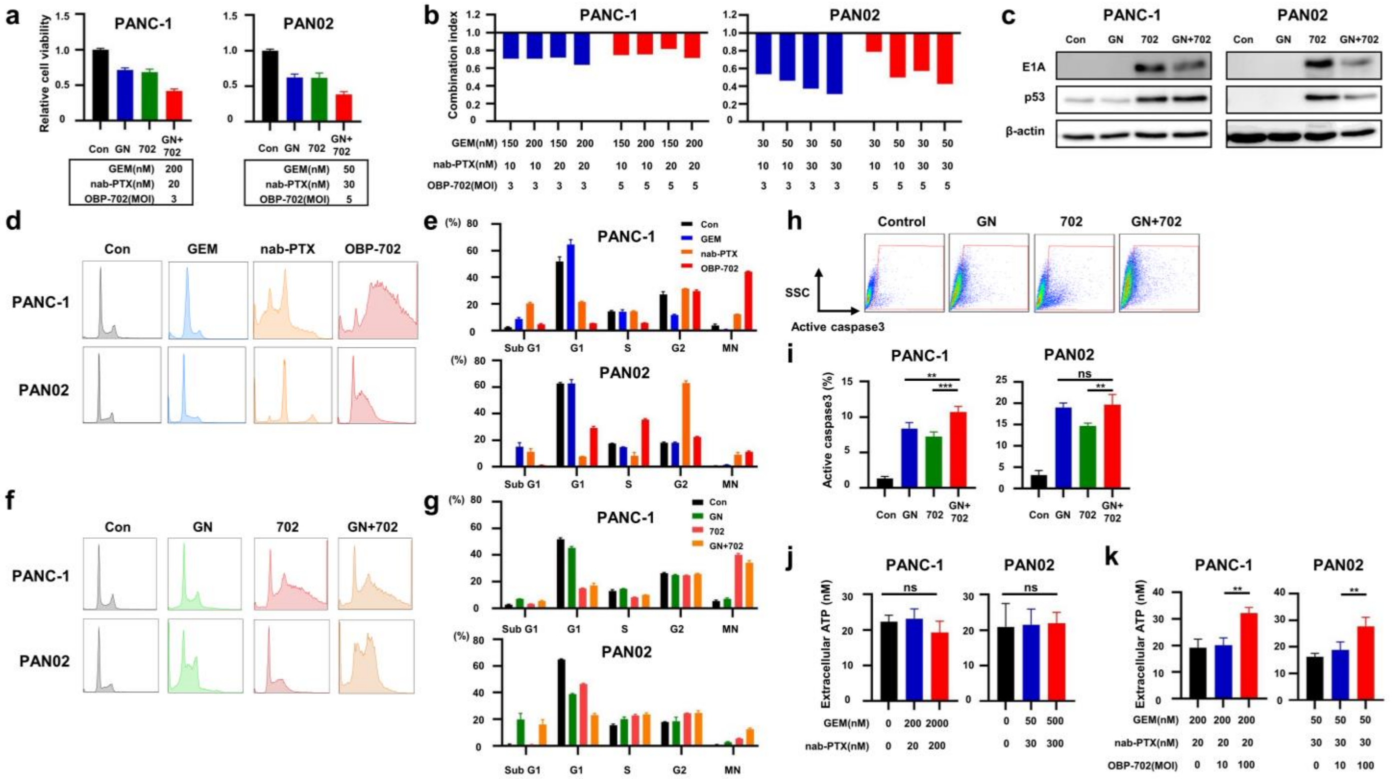

Fig. S5. Synergistic cytotoxic effects of OBP-702 with GEM and nab-PTX on human and murine pancreatic cancer cell lines

a. Viability of PANC-1 and PAN02 cells was assessed using an XTT assay 3 days after treatments with GN (GEM+nab-PTX), OBP-702, or GN+OBP-702 at the indicated doses (n=5). The percentage of viable cells relative to non-treated cells is plotted. Error bars indicate 95% confidence intervals. **b.** The combination index was assessed with CalcuSyn software (BioSoft, Cambridge, UK) based on the cell viability assay of PANC-1 and PAN02 cells 3 days after treatment with GN+OBP-702 at the indicated doses. Synergy and antagonism were defined as interaction indices of <1 and >1, respectively. **c.** Whole cell lysates of PANC-1 and PAN02 cells collected 24 hours after GN and/or OBP-702 treatments at the doses indicated in (a) were subjected to western blots of E1A, p53, and β-actin. **d,e.** PANC-1 and PAN02 cells treated with PBS, GEM (1μM), nab-PTX (100 nM), or OBP-702 (10 MOI) for 48 hours were subjected to flow cytometry for the cell cycle. Representative figures of flow cytometry are shown in (d), and the percentage of cells in each phase of sub G1, G1, S, G2, or multiple nuclei (MN) is compared among control, GEM, nab-PTX, and OBP-702 (n=3) (e). **f,g.** PANC-1 and PAN02 cells treated with PBS, GN, OBP-702, or GN+OBP-702 for 48 hours were subjected to flow cytometry for the cell cycle. Representative figures of flow cytometry are shown in (f), and the percentage of cells in each phase of sub G1, G1, S, G2, or MN are compared among control, GN, OBP-702, and GN+OBP-702 (n=3) (g). **h,i.** PANC-1 and PAN02 cells treated with PBS, GN, OBP-702, or GN+OBP-702 for 72 hours were subjected to flow cytometry for active caspase-3 (BD Biosciences). Representative figures of flow cytometry are shown in (h), and the percentage of active caspase-3-positive cells is compared among control, GN, OBP-702, and GN+OBP-702 (n=3-4) (i). **j.** Extracellular levels of ATP secreted from PANC-1 and PAN02 cells treated with GN at the indicated doses were measured with a luminescence assay 24 hours after treatment (n=4). **k.** Extracellular levels of ATP secreted from PANC-1 and PAN02 cells treated with GN+BP-702 at the indicated doses were measured with a luminescence assay 24 hours after treatment (n=4). **p* <0.05, ***p* <0.005, ****p* <0.001. Con, control; GEM, gemcitabine; nab-PTX, nab-paclitaxel; GN, GEM+nab-PTX; 702, OBP-702; ATP, adenosine triphosphate; MOI, multiplicity of infection; MN, multiple nucleus.

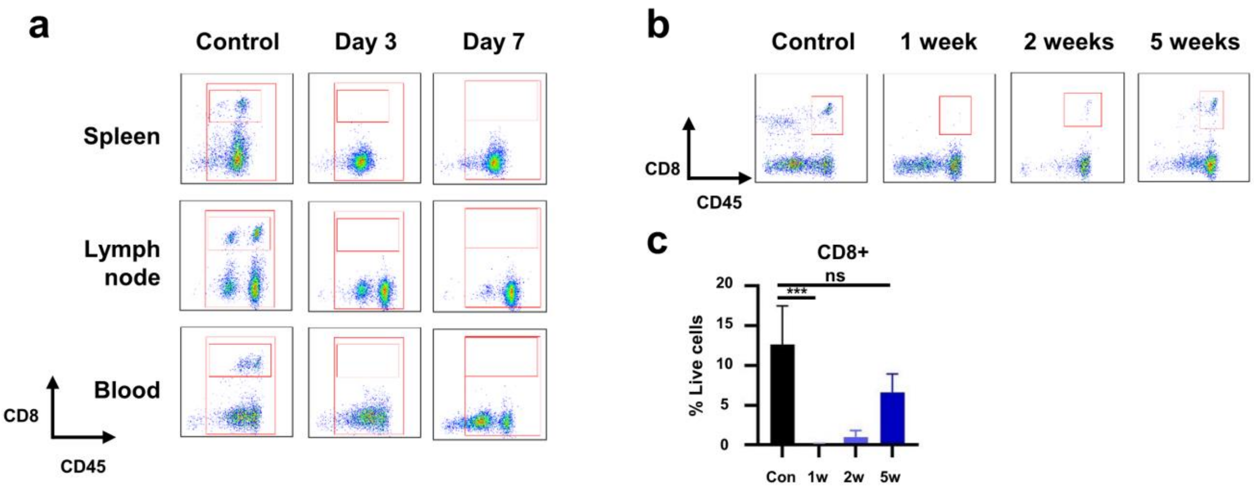

Fig. S6. Supporting data for Fig. 4 and Fig. 5

a. Representative figures of flow cytometry for CD8+ T cells in the spleen, lymph nodes, and blood. C57BL6 mice were injected with anti-CD8α antibody (20 μg) intraperitoneally and sacrificed 3 or 7 days after injection for the analysis. **b,c.** Blood collected over time 1, 2, and 5 weeks after intraperitoneal injection of anti-CD8α antibody (20 μg) was subjected to flow cytometry for CD8+ T cells. Representative figures of flow cytometry are shown in (b), and populations of CD8+ T cells are analyzed over time (n=3-5) (c). *p <0.05, **p <0.005, ***p <0.001. Con, control; GN+702, gemcitabine + nab-paclitaxel + OBP-702.

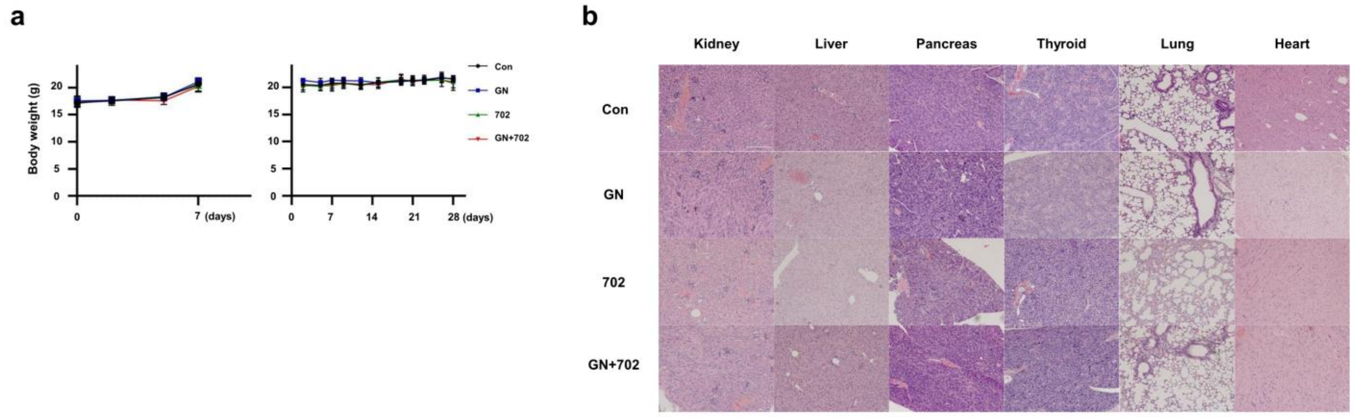

Fig. S7. Supporting data for Fig. 5

a. Body weight changes (before resection and after re-inoculation) of mice treated in the protocol of Fig. 5a.
b. Representative images of hematoxylin and eosin staining of organs (kidney, liver, pancreas, thyroid, lung, and heart) harvested after sacrifice in the protocol of Fig. 5a. Scale bar, 200 µm. Con, control; 702, OBP-702; GN; gemcitabine + nab-paclitaxel.
